# Primary central nervous system lymphoma: Predictors of response to induction therapy and patterns of progression

**DOI:** 10.1093/noajnl/vdaf082

**Published:** 2025-04-24

**Authors:** Louis Cappelli, Allison Kayne, Jennifer Newman, Ahmed Elguindy, Narendranath Epperla, Joshua D Palmer, Ubaldo Martinez Outschoorn, Pierluigi Porcu, Wenyin Shi, Iyad Alnahhas

**Affiliations:** Department of Radiation Oncology, Thomas Jefferson University, Philadelphia, Pennsylvania, USA; Department of Internal Medi cine, Thomas Jefferson University, Philadelphia, Pennsylvania, USA; Department of Neurolog y, Memorial Sloan Kettering Cancer Center, New York, New York, USA; Department of Radiation Oncology, The James Cancer Hospital at The Ohio State University Wexner Medical Center, Columbus, Ohio, USA; Division of Hematology and Hematologic Malignancies, Department of Medicine, Huntsman Cancer Institute, University of Utah, Salt Lake City, Utah, USA; Department of Radiation Oncology, The James Cancer Hospital at The Ohio State University Wexner Medical Center, Columbus, Ohio, USA; Department of Medical Oncology, Thomas Jefferson University, Philadelphia, Pennsylvania, USA; Department of Medical Oncology, Thomas Jefferson University, Philadelphia, Pennsylvania, USA; Department of Radiation Oncology, Thomas Jefferson University, Philadelphia, Pennsylvania, USA; Department of Neurology, Thomas Jefferson University, Philadelphia, Pennsylvania, USA

**Keywords:** CDKN2A, HDMTX, induction chemotherapy, PCNSL, relapse

## Abstract

**Background:**

Primary central nervous system lymphoma (PCNSL) is a rare and aggressive variant of non-Hodgkin lymphoma. While PCNSL is often sensitive to induction high-dose methotrexate (HDMTX) based chemotherapy, recurrence rates remain high, approaching 50% within 5 years. The most common molecular alterations in PCNSL include mutations in MYD88 and CD79 and CDKN2A homozygous deletion. There are no predictive or prognostic molecular markers in PCNSL.

**Methods:**

We conducted a retrospective review of 40 patients with PCNSL treated at Thomas Jefferson University and Ohio State University between 2011 and 2023. We created a clinically annotated database of patient characteristics and outcomes. For 13 patients whose paraffin-embedded tissue was available for analysis, Illumina’s Infinium Global Diversity Array with Cytogenetics was used to make copy number change calls.

**Results:**

The most commonly used induction chemotherapy regimens were HDMTX monotherapy and HDMTX with rituximab. The overall response rate to induction chemotherapy was 75%. A total of 25% had resistant disease to induction chemotherapy. The median follow-up was 20.3 months. The median progression-free survival for the entire cohort was 30.64 months (range 3.42–57.86 months); 2.56 months for the resistant group and 44.88 months for the sensitive group (*P*-value < .001). The median overall survival for the entire cohort was 64.8 months (range 41.47–88.13 months); 13.97 months for the resistant group and 81.43 months for the sensitive group (*P*-value = .046).

**Conclusions:**

The initial response to induction chemotherapy is an important prognostic factor in PCNSL. There is a need for improved predictive biomarkers of response to treatment in PNCLS.

**Key Points**

- Distant recurrences (outside of the enhancing/FLAIR borders of the initial disease) were more common than local recurrences among patients with PCNSL.
- CDKN2A deletions were seen in both sensitive and resistant PCNSL cases, underscoring the need for predictive biomarkers for HDMTX response.

**Importance of the Study**

This study provides insights into the treatment and progression of primary central nervous system lymphoma (PCNSL). By analyzing 40 patients treated with high-dose methotrexate (HDMTX)-based regimens, the research identifies initial chemotherapy response as a key predictor of survival, with sensitive cases achieving significantly longer progression-free survival (44.88 vs. 2.56 months) and overall survival (81.43 vs. 13.97 months) compared to resistant cases. The findings highlight the predominance of distant (outside of the enhancing/FLAIR borders of the initial disease) over local recurrences, emphasizing the need for strategic consolidation therapies. Additionally, molecular analysis reveals CDKN2A deletions in both sensitive and resistant cases, pointing to the need for biomarkers to predict treatment response. This work underscores the importance of personalized therapeutic strategies and advances the understanding of PCNSL.

Primary central nervous system lymphoma (PCNSL) is a rare and aggressive variant of non-Hodgkin lymphoma that primarily affects the brain, spinal cord, and eyes. PCNSL is relatively rare and represents 4%–6% of extranodal lymphomas and 4% of primary brain tumors.^1^ Most PCNSL is classified as diffuse large B-cell lymphoma (DLBCL). PCNSL belongs to the non-germinal center-activated B-cell subtype (ABC).^1^

Despite aggressive treatment, PCNSL recurrence rates remain high approaching 50% within 5 years.^2^ Given the significant recurrence rates, the treatment of PCNSL involves induction and consolidation phases. High-dose methotrexate (HDMTX) is the cornerstone of the induction regimens. Response rates to HDMTX range from 50% to 75%.^3^ Moreover, various induction regimens have been studied including HDMTX with cytarabine (AraC),^4^ RMVP (rituximab, HDMTX, vincristine, and procarbazine),^5^ MTR (HDMTX, temozolomide, and rituximab)^6^ and MATRiX (HDMTX, cytarabine, rituximab, and thiotepa)^7^ with different response rates. There is little head-to-head comparison data to guide treatment choice, and institutions typically adopt their own protocols. Similarly, there’s no consensus as to the number of cycles of HDMTX that should be used. Consolidation methods involve whole-brain radiation therapy (WBRT) and autologous stem-cell transplant (ASCT).^7^ Different conditioning regimens are used prior to ASCT that mainly include thiotepa, busulfan, and cyclophosphamide (TBC) and carmustine (BCNU)/thiotepa.^8^ WBRT carries cognitive risks, especially following HDMTX. Therefore, ASCT is favored for younger patients.

PCNSL relapses are more often local than disseminated.^9^ A recent large retrospective study showed that unifocal relapses are more common than multifocal relapses. It also showed that relapses involving deep structures, leptomeninges, and infratentorial relapses were less common.^10^ The treatment of PCNSL at recurrence is very challenging with limited high-level clinical trial data. Commonly used approaches include salvage WBRT and ASCT if not previously utilized, ibrutinib, pomalidomide and temozolamide-etoposide, doxil, dexamethasone-rituximab (TEDDI-R).^1^

The most common molecular alterations in PCNSL include mutations in MYD88 and CD79 and CDKN2A homozygous deletion.^11^ In a study of 39 PCNSL samples that were profiled using whole-genome sequencing, at the copy number level, PCNSL demonstrated significantly more CN losses in 6p21 (HLA-D locus) as well as recurrent losses in 9p21 (MTAP, CDKN2A/B) and 19p13 (CDKN2D) compared to DLBCL.^12^ In another study of 21 PCNSL samples that were analyzed using high-density single-nucleotide polymorphism, 9p21.3/CDKN2Acopyloss, was significantly more frequent in PCNSLs than in ABC-type DLBCLs. Moreover, copy gains of 3q12.3, 18q21.33, and 19q13.42 and copy losses of 6p21.33 and 6q21 were significantly more frequent in PCNSL than in DLBCL.^13^

To date, there are no biomarkers that are predictive of response to HDMTX or prognostic in PCNSL. In this study, we describe our clinical experience with patients with PCNSL. We highlight the concept of response to HDMTX as a potential guide to further induction chemotherapy and evaluate copy number changes in a subset of patients in an effort to predict response to HDMTX. We describe the patterns of progression seen in patients.

## Methods

All immunocompetent PCNSL patients ≥18 years old who were treated between 2011 and 2023 and had survival and imaging data available were included in the study. All patients had pathologic confirmation of the diagnosis. All patients had negative systemic imaging for systemic involvement of lymphoma. Relevant data from the medical charts including patient demographics, tumor laterality, treatment course, disease progression, patterns of recurrence, and survival were collected from electronic health records.

The International PCNSL collaborative group response criteria for PCNSL was used to assess treatment response using pre- and post-treatment MRI imaging.^14^ Time-to-response was calculated in days between the date of the initial scan and the date of the MRI scan showing the best response. Progression-free survival (PFS) was calculated as time in months between the date of the initial scan showing PCNSL and the date of the MRI showing progression. Overall survival (OS) was calculated as time in months between the date of the initial scan showing PCNSL and the date of death from any cause. Survival estimates were calculated using the Kaplan–Meier method. Log rank (Mantel-Cox Test) was used to assess survival differences between groups. SPSS 27 was used for statistical analyses.

**Figure 1.**
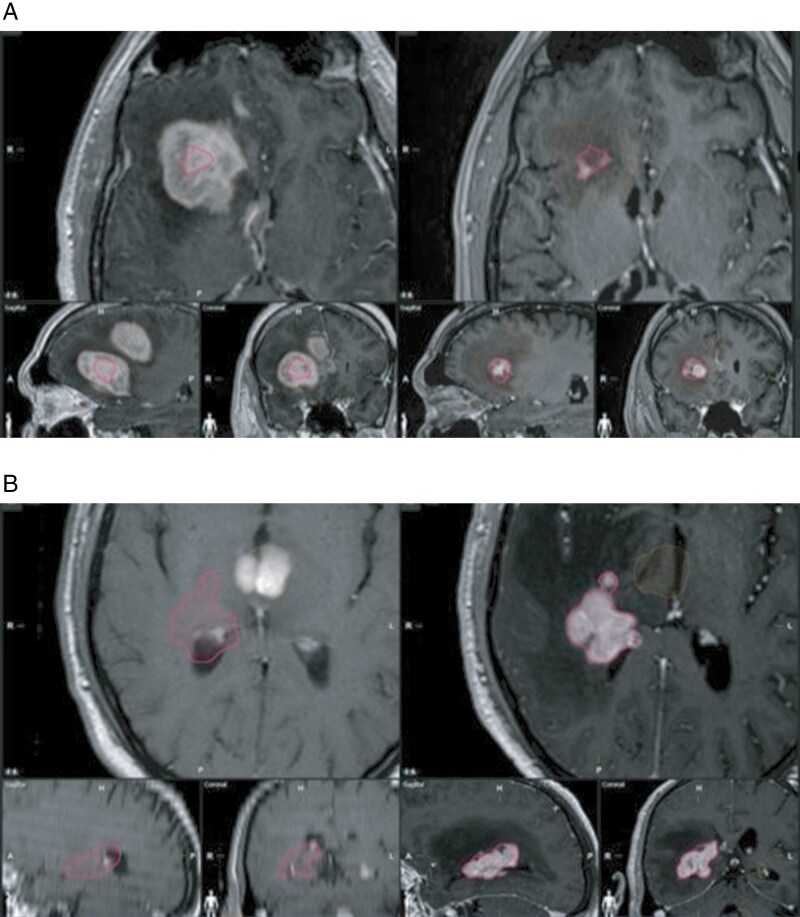
(A) Local versus distant progression of PCNSL. (A) is an example of local tumor progression on MRI T1-post contrast (left pre-HDMTX; right post-HDMTX). The black borders correlate with the lesion after progression. (B) Local versus distant progression of PCNSL. (B) is an example of distant tumor progression on MRI T1-post contrast (left pre-HDMTX; right post-HDMTX). The black borders correlate with the lesion after progression.

The pattern of recurrence was reviewed by comparing the initial diagnostic MRI to the recurrence MRI (T1 post-contrast and T2/FLAIR sequence). The pattern of recurrence was classified as either local or distant, depending on the anatomical location of the recurrent lesion. Each diagnostic MRI was reviewed by at least 2 authors to confirm the recurrence pattern. Local recurrences were classified as new lesions that overlapped with the primary enhancing lesion/FLAIR abnormality (Figure 1A). Lesions of distant recurrence were classified as those separate from the previous enhancing lesion and located outside of the initial FLAIR abnormality (Figure 1B).

In a number of patients whose paraffin-embedded tissue was available for analysis, Illumina’s Infinium Global Diversity Array with Cytogenetics (GDACyto) was used to make copy number change (CNV) calls.

## Results

### Patient Characteristics

Forty patients were included in this study, of which 21 were female (52.5%) and 19 were male (47.5%). The median age at diagnosis was 67 (range of 22–81 years). The median KPS was 70 (range of 50–100). Two patients (5%) had spine involvement at the time of diagnosis, 2 (5%) had ocular involvement, and 3 (7.5%) had CSF involvement. Twenty-two patients (55%) had multifocal disease. The treatment regimen was based on the treating oncologist’s choice. The most common induction regimen was high-dose methotrexate (HDMTX) and rituximab (16 patients) followed by HDMTX monotherapy (15 patients). Five patients received HDMTX plus AraC and 4 patients received MTR. Four patients received reduced-dose WBRT, and 1 patient received ASCT as consolidation. The median number of HDMTX cycles was 6.5 (range 1–21), with 29 patients (72.5%) receiving 3.5 g/m^2^ (range of 2.0–8.0 g/m^2^; Table 1).

**Table 1.** Patient Characteristics of the Entire Cohort

	*N* = 40	%
Sex		
Male	19	47.5%
Female	21	52.5%
Median age at diagnosis (years)	66.0 (range 22.0–81.0)	
Alive at the time of analysis		
Yes	25	62.5%
No	15	37.5%
Multifocal disease at diagnosis	22	55%
Spine involvement in diagnosis		
Yes	2	5%
No	33	82.5%
N/A	5	12.5%
Ocular involvement at diagnosis		
Yes	2	5%
No	30	75%
N/A	8	20%
CSF involvement in diagnosis		
Yes	3	7.5%
No	32	80%
N/A	5	12.5%
Median cycles of HDMTX-based induction regimen	5 (range 1–21)	
Median HDMTX dose	3.5 g/m^2^ (range: 2.0–6.5 g/m^2^)	
Induction HDMTX regimen		
HDMTX	15	37.5%
HDMTX + Rituximab	17	42.5%
Other HDMTX-based regimen*	8	20%

*Other HDMTX based regimens included: HDMTX + cytarabine,^3^ HDMTX + Rituximab + Temozolomide,^3^ HDMTX + rituximab + procarbazine,^1^ HDMTX + rituximab + procarbazine + vincristine^1^.

### Treatment Response and Survival Data

Twenty-four (60%) of the 40 patients had a complete response (CR) to initial systemic therapy, 1 (2.5%) had an unconfirmed CR (CRu), and 5 (12.5%) patients had a partial response (PR). Therefore, the overall response rate was 75% and patients in CR, CRu, and PR in aggregate (30 patients total) were considered to have sensitive disease. The median time-to-response was 74.5 days and ranged from 13 to 284 days. On the other hand, 9 (22.5%) patients had disease progression through induction chemotherapy and 1 (2.5%) patient had stable disease. These PD and SD groups (10 patients) in aggregate were considered to have resistant disease. The median number of HDMTX was 8 for the sensitive cases (range 3–21) and 3.5 for the resistant cases (range 1–8). By the time of the analysis (median follow-up 20.3 months), 25 (62.5%) patients had progression as noted on imaging. The median PFS for the entire cohort was 30.64 months (range 3.42–57.86 months). The median PFS was 2.56 months (range 1.8–3.32 months) for the resistant group and 44.88 months (30.99–58.76 months) for the sensitive group (*P* value < .001). The most common agents used at progression were HDMTX rechallenge and ibrutinib. 7 patients received salvage radiation at first progression. 15 patients were deceased by the time of analysis. The median OS for the entire cohort was 64.8 months (range 41.47–88.13 months). The median OS was 13.97 months (range 4.58–23.37 months) for the resistant group and 81.43 months (range 58.62–104.24 months) for the sensitive group (*P* value .046).

### Patterns of Progression

By the time of the analysis, 25 patients had progression as noted on imaging. 14/25 (56%) patients had distant recurrences, 8/25 (32%) had local recurrences, and 3/25 (12%) had both local and distant recurrences (Table 2).

**Table 2.** Treatment Response and Patterns of Progression Following HDMTX-Based Therapy

	*N* = 25	
Initial complete response	12	48%
Initial partial response	4	16%
Progressive disease	9	36%
Local recurrence	8	32%
Sub-ependymal spread	*n* = 1	
Distant recurrence	14	56%
Sub-ependymal spread	*n* = 1	
Local and distant recurrence	3	12%
Sub-ependymal spread	*n* = 1	
Multifocal disease patterns of recurrence	*N* = 16	
Local	5	31.25%
Distant	9	56.25%
Local and distant	2	12.5%

Of the 16 patients who had the multifocal disease at the time of diagnosis who had relapsed disease, 6 (37.5%) were refractory to induction chemotherapy, 3 (18.75%) experienced PR, 6 (37.5%) achieved CR and 1 (6.26%) achieved uCR. At the time of recurrence, 11 (68.75%) demonstrated either distant or local and distant disease, and only 5 (31.25%) showed local spread of disease. The small sample size did not allow us to determine if patients with multifocal disease at presentation had a different likelihood of having resistant disease or distant recurrences than patients with one site of disease at presentation.

### Copy Number Variation Results

20 of the 40 patients had archival tissue available. However, 7 blocks were old enough that the DNA was degraded and CNV analysis was not feasible. Thirteen patients (6 resistant cases and 7 sensitive cases) had sufficient quality DNA from paraffin-embedded tissue for GDACyto analysis. CDKN2A deletions were found in 4/6 resistant cases and 2/7 sensitive cases (Fisher’s exact test *P*-value .286). Chromosome 6q deletions were found in 2/6 resistant cases and 3/7 sensitive cases (Fisher’s exact test *P*-value 1). Chromosome 6p gains were not found in resistant cases and found in 4/7 sensitive cases (Fisher’s exact test *P*-value .07). Chr 6p25.3 amplifications have been previously reported in PCNLS.^15^

## Discussion

The treatment of PCNSL is stratified into induction and consolidation therapy phases, employing a combination of HDMTX-based regimens and subsequent strategies to sustain remission and manage disease burden. There is very limited head-to-head comparison data between the various induction regimens, and institutions typically adopt their own protocols. Similarly, there’s no consensus as to the number of cycles of HDMTX that should be used. Most recent protocols use 5–7 cycles. The IELSG32 trial demonstrated that a combination of HDMTX and cytarabine outperforms HDMTX monotherapy in terms of treatment response rates. Further enhancement of the efficacy of the induction regimen was noted with the addition of rituximab and thiotepa to HDMTX and cytarabine.^7^ Other commonly used induction regimens include R-MVP^5^ and MTR.^6^ The ANOCEF-GOELAMS prospective randomized trial showed that methotrexate, procarbazine, vincristine, and cytarabine had improved response rate, PFS, and OS compared to methotrexate and temozolomide in PCNSL patients over the age of 60.^16^

We highlight the concept of PCNSL response to induction chemotherapy as a potential guide to further treatment. Do patients really require intensive induction chemotherapy regimens if their disease is potentially sensitive to HDMTX monotherapy? There are patients in our cohort who received HDMXT monotherapy and sustained long-lasting remissions. To our knowledge, no prospective study has stratified the patients’ need for further induction therapy or consolidation therapy based on their initial response to HDMTX. In our cohort, patients with sensitive disease to induction therapy clearly have improved PFS and OS. Similarly, in a post hoc analysis of the ANOCEF-GOELAMS trial, objective response at 2 months and at the end of treatment were predictors of prolonged OS (*P* < .001 and .015, respectively).

The most common molecular alterations in PCNSL include mutations (MYD88 and CD79) and CDKN2A homozygous deletion.^11^ In addition, CN losses in 6p21 (HLA-D locus) as well as recurrent losses in 9p21 (MTAP, CDKN2A/B) and 19p13 (CDKN2D) are more common in comparison to DLBCL.^12^ We sought to assess the effect of CDKN2A homozygous deletion on sensitivity to induction chemotherapy. While the sample size was small, CDKN2A loss was observed in both sensitive and resistant PCNSL cases in our cohort. Larger prospective cohorts are needed to establish molecular predictive markers of response to induction chemotherapy in PCNSL.

Following the induction phase, consolidation therapy aims to solidify the initial response and prevent disease recurrence. The IELSG32 trial’s insights extend into this phase, revealing that WBRT and autologous stem-cell transplantation (ASCT) provide equivalent effectiveness in disease control.^7^ However, a critical distinction lies in their impact on patient’s quality of life, with ASCT showing a more favorable neurocognitive outcome profile compared to WBRT, thereby emphasizing the importance of considering long-term treatment ramifications in therapeutic decision-making. As discussed above, there is no data favoring one consolidation strategy over the other based on the response to induction chemotherapy. Even though ASCT consolidation is often recommended for younger patients with good performance status, the complications and mortality rates are not insignificant.^8^ Additionally, the role of Stereotactic Radiosurgery (SRS) in managing PCNSL, particularly in cases refractory to HDMTX, deserves attention. Studies have shown that SRS, either as an adjunct to reduced-dose WBRT or as a stand-alone treatment, can achieve notable local control, underlining the necessity of meticulous patient selection based on clinical and disease characteristics to optimize outcomes.^17,18^ These advancements underscore a paradigm shift in PCNSL management, highlighting the nuanced interplay between therapeutic efficacy and patient quality of life.

The literature suggests that PCNSL relapses are more often local than disseminated. The post hoc study on MRI characteristics of the ANOCEF-GOELAMS phase II clinical trial showed that 46% of relapses were at the initial enhancing site, 40% at a different site, and 14% at both the initial site and a different site.^19^ A recent large retrospective study evaluating relapse patterns of 559 PCNSL patients showed that unifocal relapses are more common than multifocal relapses, 65 (59%) versus 45 (41%), respectively, among 110 patients who experienced disease progression following initial therapy.^10^ Previous literature suggests that distant recurrences can be a result of seeding from occult reservoir lesions, such as the CSF and eye, or extra-CNS sites, such as the bone marrow when those sites were affected at diagnosis.^20,21^ In our cohort of 40 patients of the 25 patients, 32% had local recurrence, 56 % had distant recurrence, and 12% displayed both local and distant recurrence patterns.

### Study Limitations

This is a retrospective study and only 13 patients had enough tissue for CNV analysis. Furthermore, the reliance on MRI imaging for assessing disease progression and recurrence may not capture the complete biological behavior of PCNSL, especially in cases where imaging changes are subtle or nonspecific. Recent data suggests that ^18^F-FDG-PET/CT has improved accuracy over structural imaging in surveillance for patients with PCNSL.^22^

## Data Availability

Data from this study may be made available upon reasonable request via email to Wenyin Shi M.D., PhD, wenyin.shi@jefferson.edu

## References

[1] Schaff LR , Grommes C. Primary central nervous system lymphoma. Blood. 2022;140(9):971–979. 34699590 10.1182/blood.2020008377 PMC9437714

[2] Gavrilovic IT , Hormigo A, Yahalom J, DeAngelis LM, Abrey LE. Long-term follow-up of high-dose methotrexate-based therapy with and without whole brain irradiation for newly diagnosed primary CNS lymphoma. J Clin Oncol. 2006;24(28):4570–4574. 17008697 10.1200/JCO.2006.06.6910

[3] DeAngelis LM , Yahalom J, Thaler HT, Kher U. Combined modality therapy for primary CNS lymphoma. J Clin Oncol. 1992;10(4):635–643. 1548527 10.1200/JCO.1992.10.4.635

[4] Ferreri AJ , Reni M, Foppoli M, et al; International Extranodal Lymphoma Study Group (IELSG). High-dose cytarabine plus high-dose methotrexate versus high-dose methotrexate alone in patients with primary CNS lymphoma: A randomised phase 2 trial. Lancet. 2009;374(9700):1512–1520. 19767089 10.1016/S0140-6736(09)61416-1

[5] Omuro A , Correa DD, DeAngelis LM, et al R-MPV followed by high-dose chemotherapy with TBC and autologous stem-cell transplant for newly diagnosed primary CNS lymphoma. Blood. 2015;125(9):1403–1410. 25568347 10.1182/blood-2014-10-604561 PMC4342354

[6] Rubenstein JL , Hsi ED, Johnson JL, et al Intensive chemotherapy and immunotherapy in patients with newly diagnosed primary CNS lymphoma: CALGB 50202 (Alliance 50202). J Clin Oncol. 2013;31(25):3061–3068. 23569323 10.1200/JCO.2012.46.9957 PMC3753699

[7] Ferreri AJ , Cwynarski K, Pulczynski E, et al; International Extranodal Lymphoma Study Group (IELSG). Chemoimmunotherapy with methotrexate, cytarabine, thiotepa, and rituximab (MATRix regimen) in patients with primary CNS lymphoma: results of the first randomisation of the International Extranodal Lymphoma Study Group-32 (IELSG32) phase 2 trial. Lancet Haematol. 2016;3(5):e217–e227. 27132696 10.1016/S2352-3026(16)00036-3

[8] Alnahhas I , Jawish M, Alsawas M, et al Autologous stem-cell transplantation for primary central nervous system lymphoma: Systematic review and meta-analysis. Clin Lymphoma Myeloma Leuk. 2019;19(3):e129–e141. 30584023 10.1016/j.clml.2018.11.018

[9] Plasswilm L , Herrlinger U, Korfel A, et al Primary central nervous system (CNS) lymphoma in immunocompetent patients. Ann Hematol. 2002;81(8):415–423. 12223997 10.1007/s00277-002-0498-8

[10] Tringale KR , Scordo M, Yahalom J, et al Outcomes and relapse patterns in primary central nervous systemlymphoma: Longitudinal analysis of 559 patients diagnosed from 1983-2020. Neuro Oncol. 2024;26(11):2061–2073. 10.1093/neuonc/noae115 PMC11534311 38915246

[11] Nayyar N , White MD, Gill CM, et al MYD88 L265P mutation and CDKN2A loss are early mutational events in primary central nervous system diffuse large B-cell lymphomas. Blood Adv. 2019;3(3):375–383. 30723112 10.1182/bloodadvances.2018027672 PMC6373750

[12] Radke J , Ishaque N, Koll R, et al; ICGC MMML-Seq Consortium. The genomic and transcriptional landscape of primary central nervous system lymphoma. Nat Commun. 2022;13(1):2558. 35538064 10.1038/s41467-022-30050-y PMC9091224

[13] Chapuy B , Roemer MG, Stewart C, et al Targetable genetic features of primary testicular and primary central nervous system lymphomas. Blood. 2016;127(7):869–881. 26702065 10.1182/blood-2015-10-673236 PMC4760091

[14] Abrey LE , Batchelor TT, Ferreri AJ, et al; International Primary CNS Lymphoma Collaborative Group. Report of an international workshop to standardize baseline evaluation and response criteria for primary CNS lymphoma. J Clin Oncol. 2005;23(22):5034–5043. 15955902 10.1200/JCO.2005.13.524

[15] Zhu Q , Wang J, Zhang W, et al Whole-genome/exome sequencing uncovers mutations and copy number variations in primary diffuse large B-cell lymphoma of the central nervous system. Front Genet. 2022;13:878618. 35646048 10.3389/fgene.2022.878618 PMC9133733

[16] Omuro A , Chinot O, Taillandier L, et al Methotrexate and temozolomide versus methotrexate, procarbazine, vincristine, and cytarabine for primary CNS lymphoma in an elderly population: An intergroup ANOCEF-GOELAMS randomised phase 2 trial. Lancet Haematol. 2015;2(6):e251–e259. 26688235 10.1016/S2352-3026(15)00074-5

[17] Foreman BE , Mullikin TC, Floyd SR, et al Long-term outcomes with reduced-dose whole-brain radiotherapy and a stereotactic radiosurgery boost for primary central nervous system lymphoma. Neurooncol. Adv.. 2023;5(1):vdad097. 37706200 10.1093/noajnl/vdad097 PMC10496939

[18] Palmer JD , Bhamidipati D, Shukla G, et al Outcomes after stereotactic radiosurgery for CNS lymphoma. J Neurooncol. 2020;147(2):465–476. 32108296 10.1007/s11060-020-03444-5

[19] Tabouret E , Houillier C, Martin-Duverneuil N, et al Patterns of response and relapse in primary CNS lymphomas after first-line chemotherapy: Imaging analysis of the ANOCEF-GOELAMS prospective randomized trial. Neuro Oncol. 2017;19(3):422–429. 27994065 10.1093/neuonc/now238 PMC5464299

[20] Ambady P , Fu R, Netto JP, et al Patterns of relapse in primary central nervous system lymphoma: Inferences regarding the role of the neuro-vascular unit and monoclonal antibodies in treating occult CNS disease. Fluids Barriers CNS. 2017;14(1):16. 28577579 10.1186/s12987-017-0064-3 PMC5457655

[21] Jahnke K , Hummel M, Korfel A, et al Detection of subclinical systemic disease in primary CNS lymphoma by polymerase chain reaction of the rearranged immunoglobulin heavy-chain genes. J Clin Oncol. 2006;24(29):4754–4757. 16966685 10.1200/JCO.2006.06.7165

[22] Oh M , Cho H, Park JE, et al Enhancing prognostication and treatment response evaluation in primary CNS lymphoma with 18F-FDG PET/CT. Neuro Oncol. 2024;26(12):2377–2387. 39097777 10.1093/neuonc/noae146 PMC11630510

